# Supplementary material for: Saccharomyces boulardii (CNCM I-745) improves intestinal damage in sepsis by remodeling intestinal flora structure
Source: Front Cell Infect Microbiol. 2025 Jul 31;15:1602792. doi: 10.3389/fcimb.2025.1602792 (PMC12350329; doi:10.3389/fcimb.2025.1602792)
Supplement: Supplementary file 1 [file DataSheet1.zip › summary/summary/8_Advanced_analyse/2_PICRUSt2_result/METACYC/Group.DW4_vs_CW4_vs_BW4_vs_AW4/PICRUSt2_METACYC_Group.DW4_vs_CW4_vs_BW4_vs_AW4_diff.pdf]

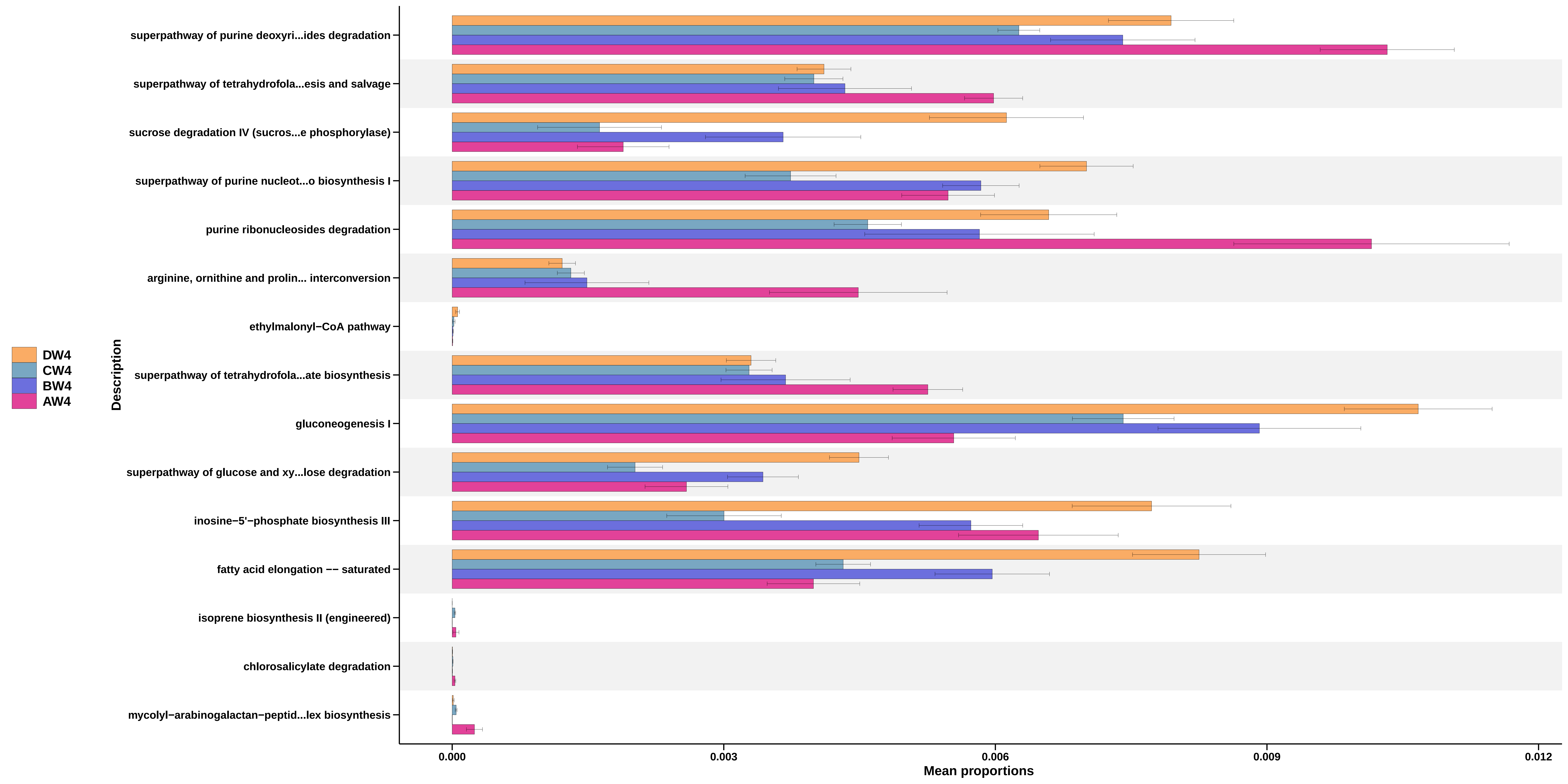

superpathway of purine deoxyri...ides degradation
superpathway of tetrahydrofola...esis and salvage
sucrose degradation IV (sucros...e phosphorylase)
superpathway of purine nucleot...o biosynthesis I
purine ribonucleosides degradation
arginine, ornithine and prolin... interconversion
ethylmalonyl−CoA pathway
superpathway of tetrahydrofola...ate biosynthesis
gluconeogenesis I
superpathway of glucose and xy...lose degradation
inosine−5'−phosphate biosynthesis III
fatty acid elongation −− saturated
isoprene biosynthesis II (engineered)
chlorosalicylate degradation
mycolyl−arabinogalactan−peptid...lex biosynthesis
Description
DW4
CW4
BW4
AW4
0.000
0.003
0.006
0.009
0.012
Mean proportions
